# Single status shows age dependent bidirectional effects in differentiated thyroid cancer

**DOI:** 10.1038/s41598-025-24280-5

**Published:** 2025-11-18

**Authors:** Xiangyi Xiao, Ruixin Zhou, Xiaolin Dou, Hui Ouyang, Xinying Li, Fada Xia, Xiwu Ouyang, Sirui Li, Chen Li

**Affiliations:** 1 Department of General Surgery, Xiangya Hospital, Central South University, Changsha, 410008 Hunan Province China; 2 National Clinical Research Center for Geriatric Disorders, Xiangya Hospital, Central South University, Changsha, 410008 Hunan Province China; 3 New York University, New York, NY 10036 USA

**Keywords:** Disease-specific survival, Differentiated thyroid cancer, Interaction effect, SEER, Single status, Cancer epidemiology, Risk factors

## Abstract

Thyroid cancer is one of the most common endocrine malignancies worldwide, and its prognosis is shaped by both tumor characteristics and social factors such as marital status. This study examined the association between single marital status and disease-specific survival (DSS) in differentiated thyroid cancer (DTC), with particular attention to its interaction with age. We analyzed 153,299 DTC cases diagnosed between 2000 and 2021 from the SEER database. Compared to married patients, single individuals were younger and exhibited larger tumors and more advanced N and M stages, though with more favorable overall TNM stages. Unadjusted Cox regression suggested a protective effect of single status (HR 0.79, 95% CI 0.72–0.87), which reversed after multivariable adjustment (HR 1.32, 95% CI 1.19–1.46). This pattern was consistent in papillary and follicular subtypes but not in Hürthle cell carcinoma. Age modified this effect: single status was protective in patients < 55 years (HR 0.89, 95% CI 0.81–0.99) but detrimental in those ≥ 55 years (HR 1.12, 95% CI 1.01–1.29). Significant interactions were observed on both multiplicative and additive scales. Fine–Gray competing risk models confirmed these findings. Overall, single status exerts an age-dependent influence on DTC prognosis and warrants consideration in risk assessment.

**Supplementary Information:**

The online version contains supplementary material available at 10.1038/s41598-025-24280-5.

## Introduction

Thyroid cancer represents one of the most prevalent endocrine malignancies globally^1^, with approximately 1.2% lifetime risk in the American population and a notable predilection for individuals under 40 years of age^2,3^. Differentiated thyroid cancer (DTC), comprising over 90% of thyroid malignancies, typically demonstrates excellent prognosis with disease-specific survival (DSS) rates exceeding 90%^4^. While conventional parameters (such as age and tumor characteristics) have been well-established as prognostic factors, emerging evidence suggests that social determinants, particularly marital status, may significantly influence patient outcomes.

Recent studies have demonstrated that marital status significantly influences cancer outcomes in DTC, with unmarried individuals (including single, separated, divorced, or widowed) generally experiencing inferior cancer-specific survival compared to their married counterparts^5,6^. Specifically, unmarried patients, particularly those who are widowed, demonstrate higher risks of advanced-stage diagnosis, suboptimal treatment adherence, and compromised survival outcomes^5^**.** However, Studies specifically assessing the impact of single status on DSS among thyroid cancer have yielded conflicting results, with protective, mixed, and harmful effects reported^5,7,8^. Furthermore, emerging evidence suggests a complex interplay between age and marital status. A recent study demonstrated that married patients exhibited a superior prognosis compared to unmarried ones in medullary thyroid cancer, with the survival benefit of marriage being more pronounced among elderly patients than their younger counterparts^9^. However, this age-dependent effect remains unexplored in DTC, despite its potential to explain the inconsistent associations between single status and survival outcomes.

To address the conflicting evidence regarding single status’s impact on DSS and to fill the critical knowledge gap concerning age-single status interaction in DTC, we conducted a large-scale population-based cohort study using the Surveillance, Epidemiology, and End Results (SEER) database. Our primary objectives were to evaluate the independent prognostic implications of single status and to systematically examine its potential age-dependent effects on DSS in DTC patients.

## Methods

### Study population

We extracted data from the Surveillance, Epidemiology, and End Results (SEER) database (17 Registries, 2000–2021) for patients diagnosed with differentiated thyroid cancer (DTC), including papillary thyroid carcinoma (PTC; ICD-O-3: 8050, 8260, 8340–8344, 8350, 8450–8460), follicular thyroid carcinoma (FTC; ICD-O-3: 8330–8332, 8335, 8339), and Hürthle cell thyroid carcinoma (HTC; ICD-O-3: 8290). Inclusion criteria comprised DTC as the first primary cancer with histological confirmation. We excluded patients with non-single/non-married status, unknown clinical information, or follow-up in less than one month. The final cohort included 153,299 patients (Supplementary Fig. 1). Due to the public nature of the SEER database, this study was exempt from review by the Institutional Review Board of Central South University.

### Variables and outcomes

We collected demographic (age, sex, race/ethnicity, household income, county type, marital status), clinicopathologic (histology, grade, tumor size, extrathyroidal extension, multifocality, nodal status, distant metastasis), and therapeutic (surgery, radiotherapy) variables. Tumor staging followed the AJCC 8th edition classification. The primary outcome was disease-specific survival (DSS), defined as the time from diagnosis to death from thyroid cancer or last follow-up.

### Statistical analysis

We generated unadjusted survival curves using Kaplan–Meier method and adjusted curves using direct adjustment based on Cox models. The impact of marital status on DSS was assessed using hazard ratios (HRs) and absolute survival differences. Given the potential prognostic heterogeneity across histological types, survival analyses were stratified accordingly. To systematically evaluate the association between marital status and DSS, we constructed several sequential Cox regression models with progressive adjustment, from basic demographic factors to comprehensive clinicopathologic and treatment variables.

To investigate the interaction effect between marital status and age, Age was categorized into two groups: less than 55 years and 55 years or older. The effect of marital status stratified by age was also assessed with HR and absolute survival differences. Additionally, interactions were evaluated on both multiplicative and additive scales. The multiplicative interaction was quantified using the ratio of hazard ratios (HR11/(HR10 × HR01)), where a value greater or less than 1 indicates that the combined effect differs from the product of individual effects. The additive interaction was assessed using the relative excess risk due to interaction (RERI = HR11 − HR10 − HR01 + 1), which measures whether the combined effect deviates from the sum of individual effects. In these calculations, HR11 represents the hazard ratio for joint exposure, while HR10 and HR01 represent the hazard ratios for each exposure independently. No significant interaction was defined as 95% confidence intervals including 1 for multiplicative scale or 0 for RERI, with confidence intervals calculated using the delta method.

A sensitivity analysis was conducted to test the robustness of the results. We repeated the analysis using the Fine and Gray competing risk model, considering non-thyroid cancer death as a competing risk. Categorical variables were summarized as frequencies and percentages, while continuous variables were presented as medians (interquartile ranges) or means (standard deviations). Between-group comparisons used χ2 or Mann–Whitney U tests as appropriate. All analyses were performed using R version 4.3.1. Significance levels are denoted as follows: ***p < 0.001, **p < 0.01, and *p < 0.05.

## Results

### Patient characteristics

A total of 153,299 patients diagnosed with DTC were identified from the SEER database for the period from 2000 to 2021 (Supplementary Fig. 1). Among these patients (Table 1), PTC was the most prevalent, accounting for 92.3%, followed by FTC at 5.6% and HTC at 2.1%. In this cohort, 112,589 patients (73.4%) were married, while 40,710 patients (26.6%) were unmarried. Single patients were significantly younger at diagnosis, with a median age of 36 years compared to 49 years for married patients (*p* < 0.001). Specifically, a higher proportion of single patients (82.6%) were aged below 55 years compared to 64.5% of married patients. Additionally, single patients were more likely to be female (78.9% vs. 74.9%) and to belong to urban populations (92.8% vs. 90.1%). Notably, while single patients presented with larger tumor sizes and a greater incidence of extrathyroidal extension as well as advanced N and M stages (all *p* < 0.001), they also had a higher proportion of earlier initial TNM stages. Concerning treatment, single patients were more likely to undergo total thyroidectomy than married patients (81.7% vs. 81.6%, *p* < 0.001), however, they also had a higher proportion of patients who did not receive any surgery (2.5% vs. 1.9%,* p* < 0.001). Furthermore, single patients were more likely to receive isotopes for radiotherapy compared to married patients (44.7% vs. 43.9%, *p* = 0.012).

**Table 1:** Characteristics of patients by marital status in differentiated thyroid cancer.

Variable	Overall, N = 153,299^1^	Married, N = 112,589^1^	Single, N = 40,710^1^	*p* value^2^
Histology				< 0.001
PTC	141,529 (92.3%)	104,027 (92.4%)	37,502 (92.1%)	
FTC	8,536 (5.6%)	6,052 (5.4%)	2,484 (6.1%)	
HTC	3,234 (2.1%)	2,510 (2.2%)	724 (1.8%)	
Age (year)	46.00 (36.00, 57.00)	49.00 (39.00, 59.00)	36.00 (26.00, 50.00)	< 0.001
Age category (year)				< 0.001
Below 55	106,289 (69.3%)	72,666 (64.5%)	33,623 (82.6%)	
Above 55	47,010 (30.7%)	39,923 (35.5%)	7,087 (17.4%)	
Sex				< 0.001
Female	116,470 (76.0%)	84,337 (74.9%)	32,133 (78.9%)	
Male	36,829 (24.0%)	28,252 (25.1%)	8,577 (21.1%)	
Race/ethnicity				< 0.001
Non-Hispanic White	97,069 (63.3%)	74,483 (66.2%)	22,586 (55.5%)	
Non-Hispanic Black	8,748 (5.7%)	4,664 (4.1%)	4,084 (10.0%)	
Non-Hispanic Asian or Pacific Islander	18,066 (11.8%)	14,041 (12.5%)	4,025 (9.9%)	
Non-Hispanic other/Unknown	1,895 (1.2%)	1,264 (1.1%)	631 (1.5%)	
Hispanic	27,521 (18.0%)	18,137 (16.1%)	9,384 (23.1%)	
Household income				< 0.001
Low	40,033 (26.1%)	29,706 (26.4%)	10,327 (25.4%)	
Middle	62,997 (41.1%)	45,551 (40.5%)	17,446 (42.9%)	
High	50,269 (32.8%)	37,332 (33.2%)	12,937 (31.8%)	
County type				< 0.001
Urban	139,256 (90.8%)	101,491 (90.1%)	37,765 (92.8%)	
Rural	14,043 (9.2%)	11,098 (9.9%)	2,945 (7.2%)	
Grade				0.231
Well differentiated; Grade I	20,619 (13.5%)	15,086 (13.4%)	5,533 (13.6%)	
Moderately differentiated; Grade II	4,217 (2.8%)	3,146 (2.8%)	1,071 (2.6%)	
Poorly differentiated; Grade III	1,270 (0.8%)	921 (0.8%)	349 (0.9%)	
Unknown	127,193 (83.0%)	93,436 (83.0%)	33,757 (82.9%)	
Tumor size				< 0.001
0–20	94,934 (61.9%)	71,878 (63.8%)	23,056 (56.6%)	
20–40	36,439 (23.8%)	25,605 (22.7%)	10,834 (26.6%)	
40–90	14,376 (9.4%)	9,573 (8.5%)	4,803 (11.8%)	
Unknown	7,550 (4.9%)	5,533 (4.9%)	2,017 (5.0%)	
Extension				< 0.001
No	127,244 (83.0%)	93,933 (83.4%)	33,311 (81.8%)	
Gross	18,620 (12.1%)	13,522 (12.0%)	5,098 (12.5%)	
Unknown	7,435 (4.8%)	5,134 (4.6%)	2,301 (5.7%)	
Multifocality				< 0.001
No	61,017 (39.8%)	44,915 (39.9%)	16,102 (39.6%)	
Yes	41,417 (27.0%)	30,966 (27.5%)	10,451 (25.7%)	
Unknown	50,865 (33.2%)	36,708 (32.6%)	14,157 (34.8%)	
N stage				< 0.001
N0	115,456 (75.3%)	87,321 (77.5%)	28,135 (69.1%)	
N1	36,745 (24.0%)	24,524 (21.8%)	12,221 (30.0%)	
Unknown	1,098 (0.7%)	744 (0.7%)	354 (0.9%)	
M stage				< 0.001
M0	149,169 (97.3%)	109,737 (97.5%)	39,432 (96.9%)	
M1	1,911 (1.2%)	1,347 (1.2%)	564 (1.4%)	
Unknown	2,219 (1.4%)	1,505 (1.3%)	714 (1.8%)	
TNM stage				< 0.001
I	133,066 (86.8%)	96,110 (85.4%)	36,956 (90.8%)	
II	12,923 (8.4%)	10,684 (9.5%)	2,239 (5.5%)	
III	1,171 (0.8%)	1,003 (0.9%)	168 (0.4%)	
IV	1,676 (1.1%)	1,361 (1.2%)	315 (0.8%)	
Unknown	4,463 (2.9%)	3,431 (3.0%)	1,032 (2.5%)	
Surgery				< 0.001
Lobectomy	23,551 (15.4%)	17,635 (15.7%)	5,916 (14.5%)	
Total thyroidectomy	125,300 (81.7%)	91,866 (81.6%)	33,434 (82.1%)	
No surgery	3,134 (2.0%)	2,127 (1.9%)	1,007 (2.5%)	
Less than lobectomy	1,314 (0.9%)	961 (0.9%)	353 (0.9%)	
Radiotherapy				0.012
Isotopes	67,626 (44.1%)	49,418 (43.9%)	18,208 (44.7%)	
Beam/Implants	2,917 (1.9%)	2,137 (1.9%)	780 (1.9%)	
None/Unknown	82,756 (54.0%)	61,034 (54.2%)	21,722 (53.4%)	
Follow-up (months)	100.00 (48.00, 160.00)	103.00 (50.00, 162.00)	91.00 (43.00, 153.00)	< 0.001
Death				< 0.001
Alive	142,499 (93.0%)	104,175 (92.5%)	38,324 (94.1%)	
DSS	2,525 (1.6%)	1,994 (1.8%)	531 (1.3%)	
Other	8,275 (5.4%)	6,420 (5.7%)	1,855 (4.6%)	

^1^n (%); Median (IQR).

^2^Pearson’s Chi-squared test; Wilcoxon rank sum test.

### Effects of marital status on DSS

**Fig. 1:**
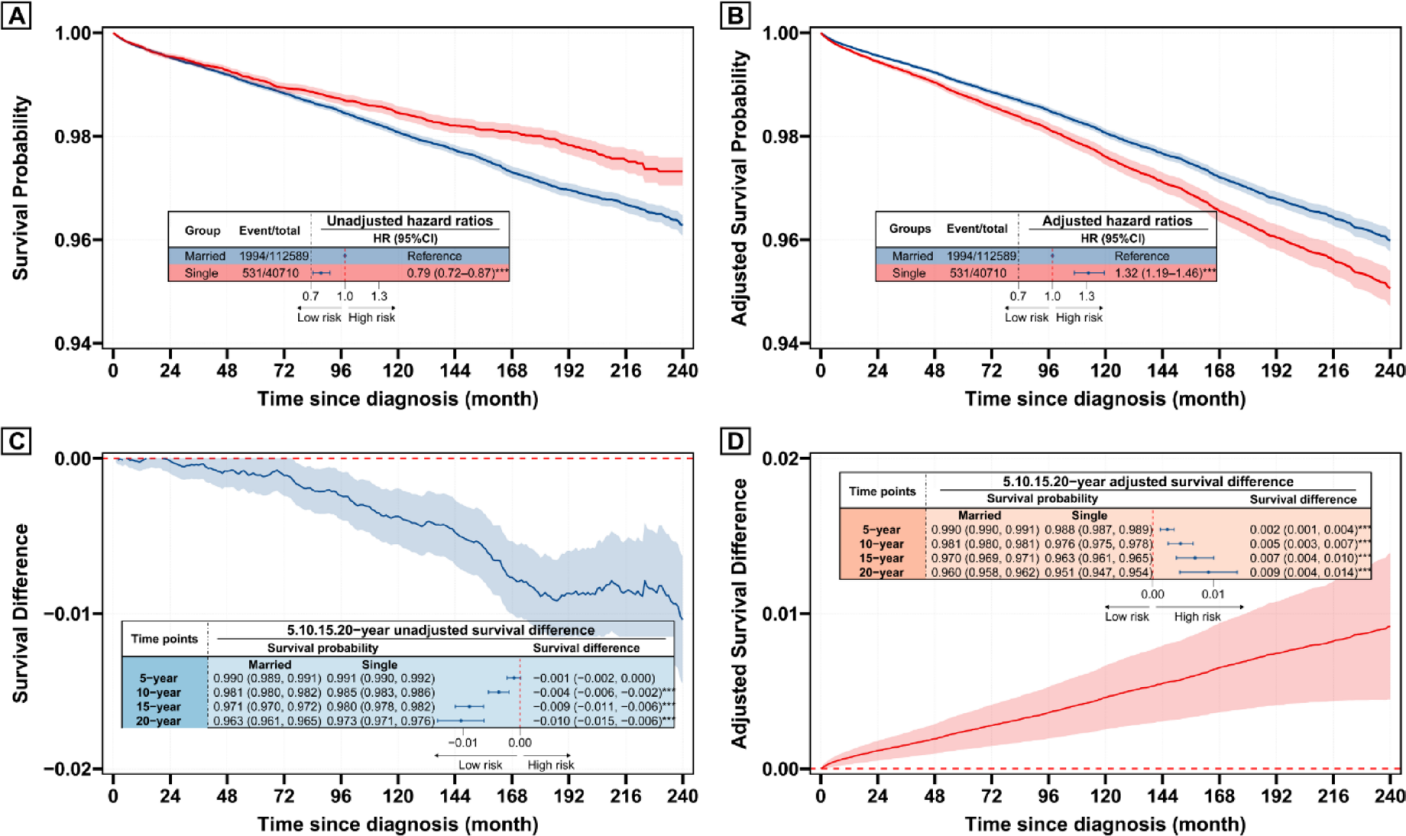
Survival curves and difference curves by marital status based on Cox model. (**A**, **C**) Unadjusted curves—survival probability (**A**) and survival differences (**C**) for single vs married patients estimated by Kaplan–Meier method. (**B**, **D**) Adjusted curves—survival probability (**B**) and survival differences (**D**) for single vs married patients derived from a multivariate Cox model adjusted for age, sex, race/ethnicity, income, county type, histology, grade, tumor size, extrathyroidal extension, multifocality, N stage, M stage, surgery, and radiotherapy.

As illustrated in Fig. 1A, Kaplan–Meier analysis showed that single patients had significantly better DSS compared to married patients (unadjusted HR 0.79, 95% CI 0.72–0.87). However, after adjusting for potential covariates, the multivariate analysis revealed a completely reversed effect, with single status identified as a risk factor for DSS (adjusted HR 1.32, 95% CI 1.19–1.46) (Fig. 1B). Absolute survival differences between single and married patients were also evaluated (Fig. 1C,D). In the unadjusted analysis, single patients consistently demonstrated a survival advantage, with higher survival probabilities over time (Fig. 1C). In contrast, after adjusting for relevant covariates, this trend reversed, and the adjusted survival of single patients gradually fell behind that of married patients over time. Notable, Stepwise adjustments in multivariate Cox models revealed that the reversal effect of marital status on DSS was primarily driven by age (Table 2), After adjusting for age, the hazard ratio (HR) for single patients increased to 1.57 (95% CI 1.42–1.72), indicating that age plays a pivotal role in determining the survival advantage. Further adjustments for other demographic (e.g., sex, race/ethnicity) and clinical variables (e.g., tumor characteristics, treatment) resulted in HRs ranging from 1.32 to 1.63. Similar results were confirmed by a sensitivity analysis using the Fine-Grey model (Supplementary Fig. 2 and Table 1).

**Table 2:** Multivariate Cox models for the effect of marital status on DSS.

Marital status	Crude model		Model1^a^		Model2^b^		Model3^c^		Model4^d^		Model5^e^	
	HRs	10-year DSS	HRs	10-year DSS	HRs	10-year DSS	HRs	10-year DSS	HRs	10-year DSS	HRs	10-year DSS
Married	Reference	0.981 (0.980, 0.982)	Reference	0.981 (0.981, 0.982)	Reference	0.981 (0.980, 0.982)	Reference	0.981 (0.980, 0.982)	Reference	0.981 (0.980, 0.982)	Reference	0.981 (0.980, 0.981)
Single	0.79 (0.72–0.87)***	0.985 (0.983, 0.986)	1.57 (1.42–1.72)***	0.972 (0.969, 0.974)	1.62 (1.47–1.79)***	0.970 (0.968, 0.973)	1.63 (1.48–1.80)***	0.970 (0.968, 0.973)	1.36 (1.23–1.50)***	0.976 (0.974, 0.977)	1.32 (1.19–1.46)***	0.976 (0.974, 0.978)

DSS, disease-special survival;

***p < 0.001;

^a^Adjusted for age;

^b^Adjusted for age, sex and race/ethnicity;

^c^Adjusted for age, sex, race/ethnicity, income and county type;

^d^Adjusted for age, sex, race/ethnicity, income, county type, histology type, grade, tumor size, extrathyroidal extension, multifocality, N stage and M stage;

^e^Adjusted for age, sex, race/ethnicity, income, county type, histology type, grade, tumor size, extrathyroidal extension, multifocality, N stage and M stage, surgery and radiotherapy.

### Effects of marital status on DSS across different histological types

**Fig. 2:**
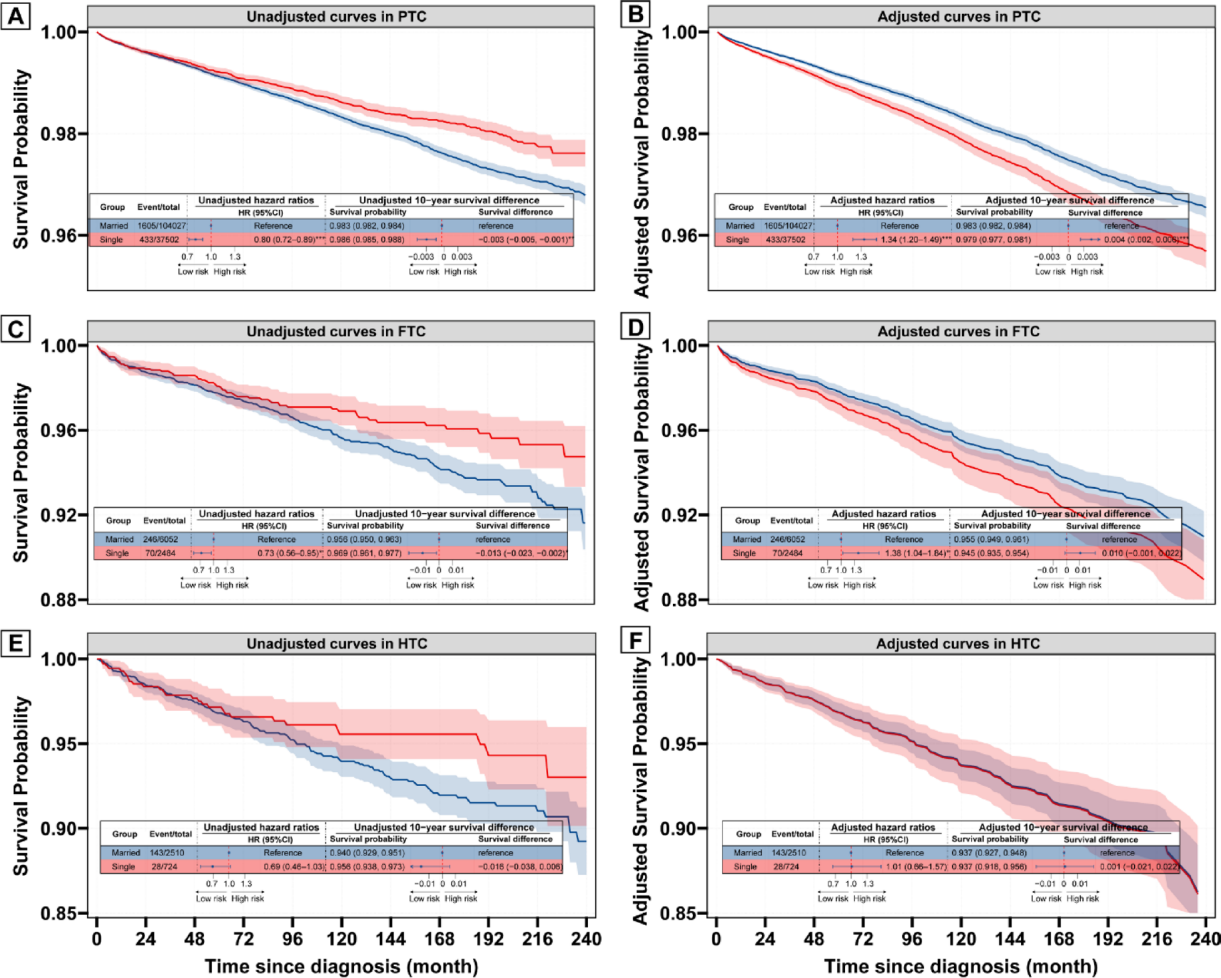
Survival curves according to marital status stratified by histology based on Cox model. (**A**, **C**, **E**) Unadjusted curves—survival probability for single vs married patients estimated by Kaplan–Meier method for PTC (**A**), FTC (**C**), and HTC (**E**) patients, respectively. (**B**, **D**, **F**) Adjusted curves—survival probability for single vs married patients derived from a multivariate Cox model adjusted for age, sex, race/ethnicity, income, county type, grade, tumor size, extrathyroidal extension, multifocality, N stage, M stage, surgery, and radiotherapy for PTC (**B**), FTC (**D**), and HTC (**F**) patients, respectively.

In the subgroup analysis of different histological types of DTC (Fig. 2), Kaplan–Meier analysis demonstrated that single status was a protective factor for DSS in PTC patients, with an unadjusted hazard ratio (HR) of 0.80 (95% CI 0.72–0.89) and a 10-year unadjusted survival difference of − 0.3% (95% CI − 0.5% to − 0.1%) (Fig. 2A). However, the adjusted analysis revealed a reversal, identifying single status as a risk factor for DSS, with an adjusted HR of 1.34 (95% CI 1.20–1.49) and a 10-year adjusted survival difference of 0.4% (95% CI 0.2–0.6%) (Fig. 2B). Similarly, for FTC, single status was associated with a protective effect in the Kaplan–Meier analysis (unadjusted HR 0.73, 95% CI 0.56–0.95) (Fig. 2C). This effect was reversed in the multivariate analysis, where single status emerged as a risk factor, with an adjusted HR of 1.38 (95% CI 1.04–1.84) (Fig. 2D). In contrast, for HTC, Kaplan–Meier analysis suggested a trend toward a protective effect of single status, but this was not statistically significant (unadjusted HR 0.69, 95% CI 0.46–1.03) (Fig. 2E). After adjustment, the multivariate Cox analysis showed no meaningful difference in DSS between single and married patients (adjusted HR 1.01, 95% CI 0.66–1.57) (Fig. 2F). Consistent patterns across all three histological subtypes were observed in sensitivity analyses using the Fine-Gray model (Supplementary Fig. 3).

### Interaction effect between age and marital status

**Fig. 3:**
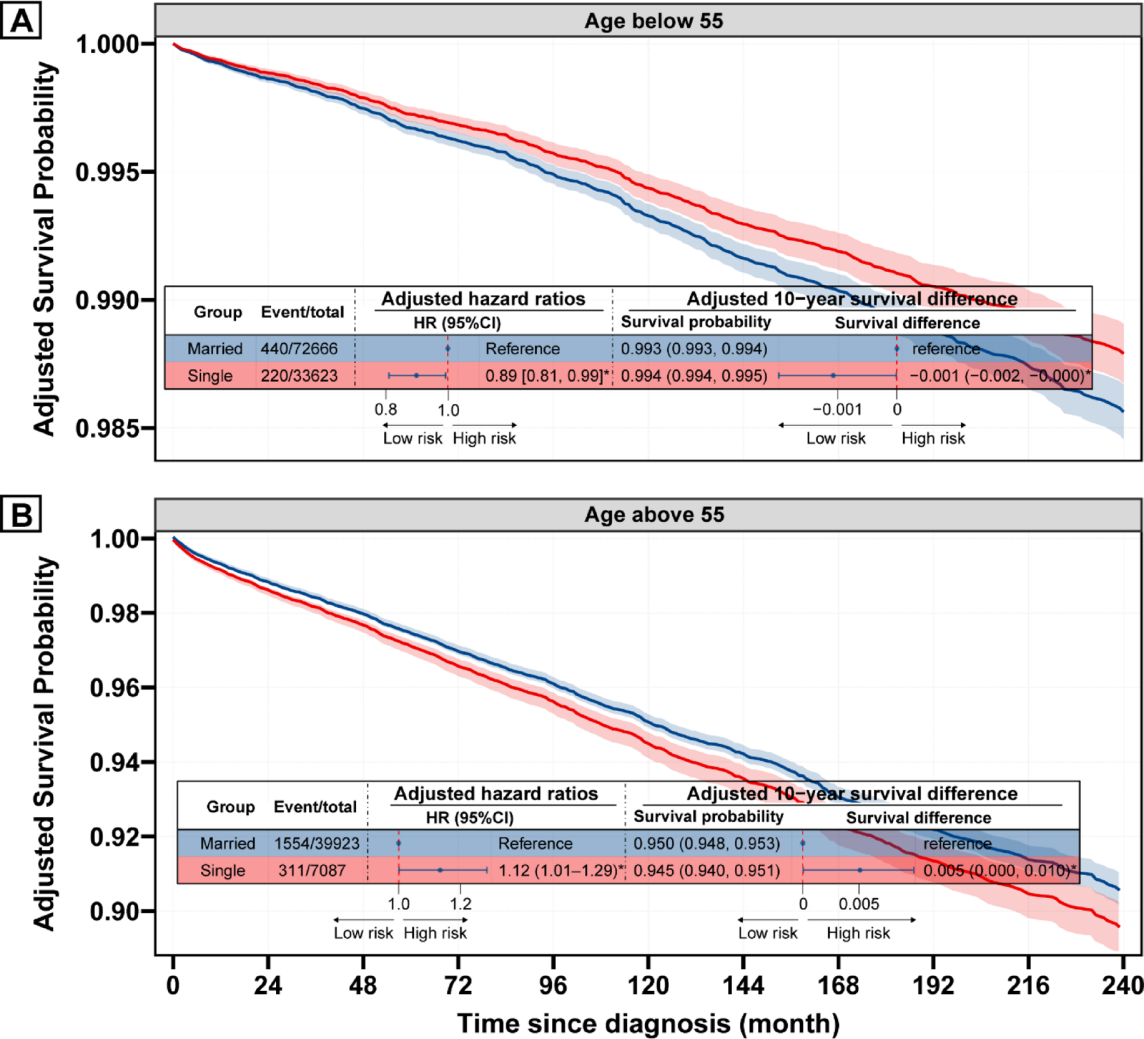
Survival curves according to marital status stratified by age based on Cox model. Survival probability for single vs married patients derived from a multivariate Cox model adjusted for sex, race/ethnicity, income, county type, histological type, grade, tumor size, extrathyroidal extension, multifocality, N stage, M stage, surgery, and radiotherapy for patients below 55 years (**A**) and patients above 55 years (**B**).

As shown in the adjusted survival curves stratified by age (Fig. 3), In younger patients (age < 55), single patients demonstrated better adjusted DSS than married patients (HR 0.89, 95% CI 0.81–0.99), with a 10-year survival difference of − 0.001 (95% CI − 0.002 to − 0.000). Conversely, in older patients (age ≥ 55), single status became a risk factor for poorer DSS (HR 1.12, 95% CI 1.01–1.29), with a 10-year survival difference of 0.005 (95% CI 0.000 to 0.010). This reversal at age 55 underscores the interaction between age and marital status. Interaction analysis (Table 3) revealed a significant positive interaction between single status and age, with a multiplicative scale of 1.26 (95% CI 1.03–1.54) and additive measures showing a RERI of 0.82 (95% CI 0.01–1.63). Notably, the interaction analysis using the Fine-Gray model yielded consistent results (Supplementary Fig. 4 and Table 2).

**Table 3:** Adjusted interaction analysis between different marital status and age groups based on Cox model.

	Married	Single	Effect of marital status within the strata of age
	HR [95% CI]	HR [95% CI]	HR [95% CI]
Age below 55	1 [Reference]	0.89 [0.81, 0.99]	0.89 [0.81, 0.99]
Age above 55	5.86 [5.25, 6.54]	6.57 [5.65, 7.63]	1.12 [1.01, 1.29]
Effect of age within the strata of marital status	5.86 [5.25, 6.54]	7.36 [6.17, 8.78]	
Multiplicative scale	1.26 [1.03, 1.54]		
RERI	0.82 [0.01, 1.63]		

Adjusted interaction analysis derived from a multivariable Cox model adjusted for covariates of sex, race/ethnicity, income, county type, histology type, grade, tumor size, extrathyroidal extension, multifocality, N stage and M stage, surgery and radiotherapy.

RERI, relative excess risk due to interaction.

## Discussion

Although marital status is widely recognized as a significant factor influencing survival and prognosis in thyroid cancer, the role of single status in differentiated thyroid cancer (DTC) remains controversial, and the interaction between marital status and age has been insufficiently addressed. This study aimed to evaluate the independent prognostic implications of single status and systematically examine its potential age-dependent effects on disease-specific survival (DSS) in DTC patients. Our findings indicated that single status initially appeared to be a protective factor for DSS among DTC patients. However, after adjusting for potential confounders, this effect shifted to a negative association with DSS. This intriguing reversal was observed across different histological types of DTC, particularly in papillary thyroid carcinoma (PTC) and follicular thyroid carcinoma (FTC), while in Hurthle cell carcinoma (HTC), the effect of single status was not statistically significant. Furthermore, we uncovered the bidirectional effect of single status across different age groups. Specifically, for patients under the age of 55, single status was associated with improved survival outcomes. In contrast, for those aged 55 and older, being single became a risk factor for poorer DSS. Additionally, we revealed a significant positive interaction between single status and age, highlighting the modifying role of age in influencing survival outcomes for DTC patients with single status. These findings underscore the need for further research into the interplay of marital status and age-related factors in cancer prognosis.

Typically, being single or unmarried is regarded as a risk factor for poor disease prognosis—a trend observed across various cancers, including colorectal, gallbladder, cervical, pancreatic, lung, breast, and prostate cancers^10–14^. However, our univariate analysis indicates that single status, rather than marital status, is associated with improved DSS in DTC^5^. Surprisingly, after adjusting for age in the Cox model, the hazard ratio (HR) for single patients increased to 1.57 (95% CI 1.42–1.72). Further adjustments for other demographic factors and clinical variables resulted in HRs ranging from 1.32–1.63. The contrasting roles of single status between univariate and multivariate analyses suggest age may modify its true impact. Notably, our multivariate findings contradict a recent study by Li et al., which suggested that single patients with thyroid cancer experience significantly better DSS and overall survival (OS)^7^. This discrepancy arises because their multivariate analysis did not take age into account. This underscores the complexity of assessing the implications of marital status on prognosis and emphasizes the critical importance of incorporating age-related factors in such analyses.

This interesting reversal between univariate and multivariate analysis remains consistent when we examine the influence of single status across different subtypes of DTC, particularly in PTC and FTC. However, single status does not appear to be an influential factor in HTC. One possible explanation for this finding is the relatively rare incidence of HTC, with rates of 2.2% in the married group and 1.8% in the single group, leading to an insufficient sample size for statistical significance. Additionally, the significantly more aggressive clinical course and poorer prognosis associated with HTC may diminish the impact of single status on survival outcomes, although this remains a subject of debate^15,16^. While previous studies have explored the association between marital status and survival in DTC^5^, our research is the first to thoroughly consider the heterogeneity among subgroups and investigate the implications of single status specifically. This nuanced approach enhances our understanding of how marital status can vary in its impact on survival across different DTC subtypes.

Notably, our research is the first to uncover the bidirectional modifying effect of age on single status in differentiated thyroid cancer (DTC). Specifically, for patients under the age of 55, single status is associated with better survival outcomes, while for those aged 55 and older, it becomes a significant risk factor. This reversal is further confirmed by the analysis of interaction effects. This phenomenon can be interpreted from two distinct perspectives. For older adults, marital status often provides crucial behavioral and psychological support, particularly as they may be more vulnerable to declining social cohesion^17^. Compared to single individuals, married patients tend to demonstrate better disease management, benefiting from early diagnosis, treatment adherence, and emotional support from spouses and family members^14,18–20^. In contrast, older single patients may lack these advantages, leading to increased stress and a higher likelihood of depression^17,21^. Physiologically, chronic stress and depression can trigger elevated cortisol secretion and disrupt its circadian rhythm^22,23^, which is associated with increased neutrophil extracellular traps and reduced T cell infiltration, ultimately promoting tumor progression and metastasis^24,25^. Other stress-related neurotransmitters, such as dopamine and norepinephrine, have also been implicated in altering the tumor microenvironment^26^. For younger patients, while marital status can offer similar support, they may not fully benefit from the early stages of marriage amid rising societal pressures. Some research suggests that marriage can create additional burdens through role strain and parental stress, potentially diminishing its benefits^27–30^. Furthermore, depression levels tend to be relatively lower among single young adults compared to their older counterparts^31^, which may further explain the differences in survival outcomes between these age groups. Yet, there is a growing body of evidence indicating that single or childless status is associated with poorer health habits and greater social vulnerabilities^32–34^. This controversy suggests that while marriage-related social support mechanisms are significant, they may not be the sole determinants of prognosis in younger single patients. Beyond social support, variations in sex hormone levels may underlie the bidirectional effects of marital status across age groups. Estrogen has been shown to upregulate ERα expression in thyroid cancer and to promote tumor proliferation through the RAS/RAF/MAPK/ERK pathway^35,36^. Higher estradiol levels in younger married patients may therefore exacerbate tumor progression^37^. In addition, cancer-related stress in this population could further amplify hormonal fluctuations^38^. Childbearing represents another factor of interest in this context. Although pregnancy induces transient hormonal changes that may increase the short-term risk of DTC recurrence, the generally favorable prognosis of DTC suggests that its long-term impact on survival is negligible^39,40^. Therefore, childbirth is unlikely to represent a major pathway through which marital status influences survival outcomes in DTC.

Importantly, this bidirectional modifying effect of age partially explains why single status appears protective overall. Unlike other cancers, DTC has a younger demographic structure, with 69.3% of patients being under 55 years old. As a result, the positive effects of single status in younger patients can obscure its negative effects in older individuals during univariate analyses. Additionally, this bidirectional effect provides indirect support for the revision of the age cutoff from 45 to 55 years in the eighth edition of the AJCC/TNM cancer staging system. This revision suggests that 55 years is a critical threshold for distinguishing between stage I and stage II, correlating with significantly different 10-year disease-specific survival rates of 99.1% versus 89.2%, respectively^41^.

Furthermore, the distinct survival patterns observed in single individuals at different age stages in differentiated thyroid cancer (DTC) underscore the significant impact of psychosocial factors on patient outcomes. Understanding these dynamics can inform clinical strategies and social welfare policies aimed at improving prognosis. If the survival benefits for older adults with DTC stem from marriage-related social support mechanisms, a practical approach would be to replicate this support within the community—such as promoting early diagnosis and proactive disease surveillance. Additionally, negative mental states, including stress and depression, appear to play a critical role in the relationship between single status and survival^42^. Therefore, it is essential for healthcare providers to screen for mental health conditions within a single population and to implement positive management strategies for those experiencing poor mental well-being, particularly among older individuals. For younger patients, the potential burdens associated with marriage may limit its benefits, highlighting the need for government initiatives to enhance social welfare and security related to marriage and child-rearing^29,30^. It is also important to note that the positive effects of being single in younger individuals with DTC may be unique but transient. Thus, future research should consider the role of single status in other diseases and its long-term implications on health outcomes.

Several limitations need to be noted in the present study. First, our analysis relies on the SEER database, and its retrospective nature may introduce inherent biases. Second, while we hypothesize that mental health status could be a significant factor influencing the relationship between single status and DTC survival, we are unable to quantify its confounding effect due to the lack of relevant data in the SEER database, such as PHQ-9 scores. Third, marital or single status can change over the course of the follow-up period, and some patients may cohabitate with a partner without formal marriage. Such variations may undermine the accuracy of our results. Despite these limitations, our research is the first to uncover the association between single status and survival in DTC, demonstrating this effect across different subgroups. Additionally, we have calculated the interaction effects between age and single status using both Cox and Fine-Gray models, which further reinforce the robustness of our findings.

In conclusion, this study found the complex interaction between single status and age in patients with DTC. While single status initially appeared to confer a survival advantage in younger patients, it was identified as a risk factor for poorer outcomes in patients aged 55 and older. These findings underscore the necessity of considering age when assessing the implications of marital status on cancer prognosis.

## Supplementary Information

Below is the link to the electronic supplementary material.

Supplementary Material 1

## Data Availability

The data used in this study were obtained from the SEER (Surveillance, Epidemiology, and End Results) Program database of the U.S. National Cancer Institute. The SEER data are publicly available upon request and can be accessed at: https://seer.cancer.gov/data/. Researchers can apply for access through the SEER Data Use Agreement process.

## Abbreviations

DSS: Disease-specific survival
DTC: Differentiated thyroid cancer
SEER: Surveillance, epidemiology, and end results
HRs: Hazard ratios
PTC: Papillary thyroid carcinoma
PTC: Follicular thyroid carcinoma
HTC: Hurthle cell carcinoma
