# Supplementary material for: Single status shows age dependent bidirectional effects in differentiated thyroid cancer
Source: Sci Rep. 2025 Nov 18;15:40537. doi: 10.1038/s41598-025-24280-5 (PMC12627438; doi:10.1038/s41598-025-24280-5)
Supplement: Supplementary file 1 — Supplementary Material 1 [file 41598_2025_24280_MOESM1_ESM.docx]

**Supplementary Materials**

**Single status shows age dependent bidirectional effects in differentiated thyroid cancer**

Xiangyi Xiao^1,2#^, Ruixin Zhou^1,2#^, Xiaolin Dou^1,2^, Hui Ouyang^1,2^, Xinying Li^1,2^, Fada Xia^1,2^, Xiwu Ouyang^1,2^, Sirui Li^3^*, Chen Li^1,2^*

1. Department of General Surgery, Xiangya Hospital, Central South University, Changsha

410008, Hunan Province, China

2. National Clinical Research Center for Geriatric Disorders, Xiangya Hospital, Central

South University, Changsha 410008, Hunan Province, China

1. New York University, New York, New York 10036, USA

#The authors have contributed equally to this article as co-first authors.

*The authors have contributed equally to this article as co-corresponding authors.

**Materials of Contents**

| **Order** | **Title** | **Page** |
| --- | --- | --- |
| Supplementary Figure1 | Flowchart of patient selection. | 1 |
| Supplementary Figure2 | Cumulative incidence curves and survival difference curves by marital status based on Fine–Gray model. | 2 |
| Supplementary Figure3 | Cumulative incidence curves according to marital status stratified by histology based on Fine-Gray model. | 3 |
| Supplementary Figure4 | Cumulative incidence curves according to marital status stratified by age based on the Fine-Gray model. | 4 |
| Supplementary Table1 | Multivariate Fine-Gray models for the effect of Marital status. | 5 |
| Supplementary Table2 | Adjusted interaction analysis between different marital status and age groups based on Fine-Gray model | 6 |

**Supplementary Figure1.** Flowchart of patient selection.


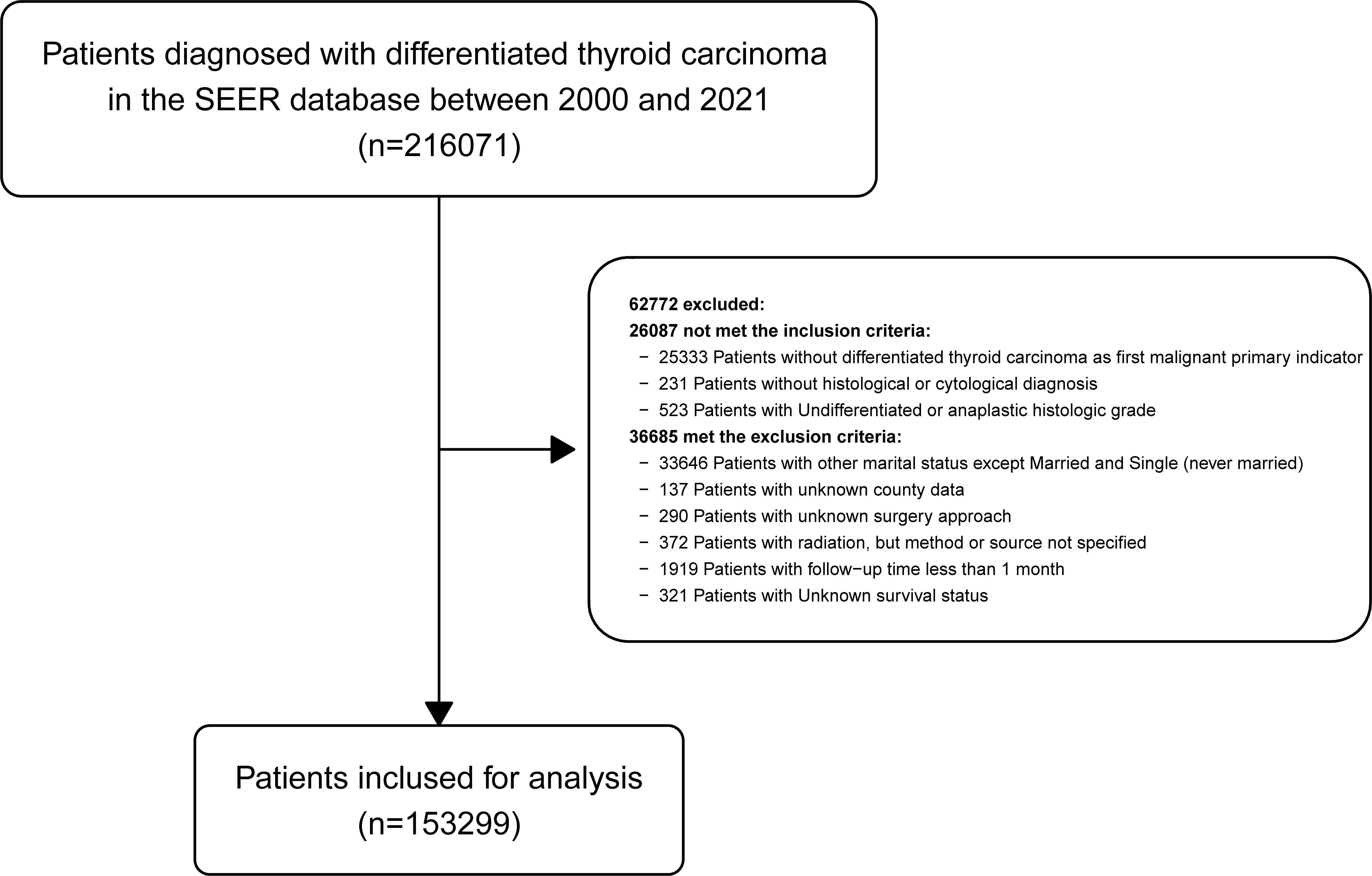


**Abbreviations:** SEER, Surveillance, Epidemiology, and End Results

**Supplementary** **Figure2.** Cumulative incidence curves and survival difference curves by marital status based on Fine–Gray model.

**Note：**

(A, C) Unadjusted curves — cumulative incidence (A) and cumulative incidence differences (C) for single vs married patients estimated without adjustment.

(B, D) Adjusted curves — cumulative incidence (B) and cumulative incidence differences (D) for single vs married patients derived from a multivariate Fine–Gray model adjusted for age, sex, race/ethnicity, income, county type, histology, grade, tumor size, extrathyroidal extension, multifocality, N stage, M stage, surgery, and radiotherapy.

**Abbreviations:** HR, hazard ratio

**Supplementary Figure3.** Cumulative incidence curves according to marital status stratified by histology based on Fine-Gray model.

**Note：**

(A, C, E) Unadjusted curves — cumulative incidence for single vs married patients estimated without adjustment for PTC (A), FTC (C), and HTC (E) patients, respectively.

(B, D, F) Adjusted curves — cumulative incidence for single vs married patients derived from a multivariate Fine–Gray model adjusted for age, sex, race/ethnicity, income, county type, grade, tumor size, extrathyroidal extension, multifocality, N stage, M stage, surgery, and radiotherapy for PTC (B), FTC (D), and HTC (F) patients, respectively.

**Abbreviations:** PTC, papillary thyroid carcinoma; FTC, follicular thyroid carcinoma; HTC, Hurthle cell carcinoma; HR, hazard ratio

**SupplementaryFigure4.** Cumulative incidence curves according to marital status stratified by age based on the Fine-Gray model.





**Note：**

Cumulative incidence of outcomes for single vs married patients derived from a multivariate Fine-Gray model adjusted for sex, race/ethnicity, income, county type, histological type, grade, tumor size, extrathyroidal extension, multifocality, N stage, M stage, surgery, and radiotherapy for patients below 55 years (A) and patients above 55 years (B).

**Abbreviations:** HR, hazard ratio

**Supplementary Table 1.** Multivariate Fine-Gray models for the effect of Marital status.

| **^Marital status^** | **Crude model** | | **Model1^a^** | | **Model2^b^** | | **Model3^c^** | | **Model4^d^** | | **Model5^e^** | |
| --- | --- | --- | --- | --- | --- | --- | --- | --- | --- | --- | --- | --- |
|  | ^HRs^ | ^10-year DSS^ | ^HRs^ | ^10-year DSS^ | ^HRs^ | ^10-year DSS^ | ^HRs^ | ^10-year DSS^ | ^HRs^ | ^10-year DSS^ | ^HRs^ | ^10-year DSS^ |
| **^Married^** | ^Reference^ | ^0.019 (0.018, 0.019)^ | ^Reference^ | ^0.017 (0.016, 0.018)^ | ^Reference^ | ^0.017 (0.017, 0.018)^ | ^Reference^ | ^0.017 (0.016, 0.018)^ | ^Reference^ | ^0.017 (0.017, 0.018)^ | ^Reference^ | ^0.017 (0.017, 0.018)^ |
| **^Single^** | ^0.79 (0.72–0.87)***^ | ^0.015 (0.014, 0.016)^ | ^1.48 (1.34–1.63)***^ | ^0.025 (0.023, 0.027)^ | ^1.52 (1.38–1.68)***^ | ^0.026 (0.024, 0.028)^ | ^1.53 (1.38–1.69)***^ | ^0.026 (0.024, 0.028)^ | ^1.24 (1.11–1.38)***^ | ^0.021 (0.019, 0.022)^ | ^1.24 (1.11–1.38)***^ | ^0.021 (0.020, 0.023)^ |

^a^Adjusted for age;

^b^Adjusted for age, sex and race/ethnicity;

^c^Adjusted for age, sex, race/ethnicity, income and county type;

^d^ Adjusted for age, sex, race/ethnicity, income, county type, histology type, grade, tumor size, extrathyroidal extension, multifocality, N stage and M stage;

^e^ Adjusted for age, sex, race/ethnicity, income, county type, histology type, grade, tumor size, extrathyroidal extension, multifocality, N stage and M stage, surgery and radiotherapy;

**Abbreviations:** HRs, hazard ratios; DSS, disease-special survival.

**Supplementary Table 2.** Adjusted interaction analysis between different marital status and age groups based on Fine-Gray model.

|  | Married | Single | Effect of age within the strata of marital status |
| --- | --- | --- | --- |
|  | HR [95% CI] | HR [95% CI] | HR [95% CI] |
| Age below 55 | 1 [Reference] | 0.89 [0.82, 0.98] | 0.89 [0.82, 98] |
| Age above 55 | 5.04 [4.5, 5.65] | 5.61 [4.77, 6.59] | 1.12 [1.00, 1.28] |
| Effect of age within the strata of marital status | 5.04 [4.5, 5.65] | 6.27 [5.21, 7.56] |  |
| Multiplicative scale | 1.24 [1.01, 1.54] |  |  |
| RERI | 0.67 [0.3, 1.48] |  |  |

**Note:** Adjusted interaction analysis derived from a multivariable Fine-Gray model adjusted for covariates of sex, race/ethnicity, income, county type, histology type, grade, tumor size, extrathyroidal extension, multifocality, N stage and M stage, surgery and radiotherapy.

**Abbreviations:** RERI, relative excess risk due to interaction.
